# Crowdsensing IoT Architecture for Pervasive Air Quality and Exposome Monitoring: Design, Development, Calibration, and Long-Term Validation

**DOI:** 10.3390/s21155219

**Published:** 2021-07-31

**Authors:** Saverio De Vito, Elena Esposito, Ettore Massera, Fabrizio Formisano, Grazia Fattoruso, Sergio Ferlito, Antonio Del Giudice, Gerardo D’Elia, Maria Salvato, Tiziana Polichetti, Paolo D’Auria, Adrian M. Ionescu, Girolamo Di Francia

**Affiliations:** 1 ENEA CR-Portici, TERIN-FSD Division, P. le E. Fermi 1, 80055 Portici, Italy; ettore.massera@enea.it (E.M.); fabrizio.formisano@enea.it (F.F.); grazia.fattoruso@enea.it (G.F.); sergio.ferlito@enea.it (S.F.); antonio.delgiudice@enea.it (A.D.G.); gerardo.delia@enea.it (G.D.); maria.salvato@enea.it (M.S.); tiziana.polichetti@enea.it (T.P.); girolamo.difrancia@enea.it (G.D.F.); 2 ARPA Campania, Via Vicinale Santa Maria del Pianto Centro Polifunzionale, Torre 1, 80143 Napoli, Italy; p.dauria@arpacampania.it; 3 NanoLab, EPFL-Ecole Politechnique Federal de Lausanne, 1015 Lausanne, Switzerland; adrian.ionescu@epfl.ch

**Keywords:** IoT AQ nodes, sensor network, calibration, air quality monitoring, machine learning

## Abstract

A pervasive assessment of air quality in an urban or mobile scenario is paramount for personal or city-wide exposure reduction action design and implementation. The capability to deploy a high-resolution hybrid network of regulatory grade and low-cost fixed and mobile devices is a primary enabler for the development of such knowledge, both as a primary source of information and for validating high-resolution air quality predictive models. The capability of real-time and cumulative personal exposure monitoring is also considered a primary driver for exposome monitoring and future predictive medicine approaches. Leveraging on chemical sensing, machine learning, and Internet of Things (IoT) expertise, we developed an integrated architecture capable of meeting the demanding requirements of this challenging problem. A detailed account of the design, development, and validation procedures is reported here, along with the results of a two-year field validation effort.

## 1. Introduction

Air quality (AQ) is one of the main factors influencing quality of life in cities [1]. Rural areas may also be affected due to peculiar geographic conditions and associated local climate dynamics that may negatively affect dispersion. Unfortunately, air pollutants are increasingly and reliably associated with several medical conditions, ranging from easy manageable and temporary illnesses to severe and seriously harmful diseases [2]. As a consequence, the number of premature deaths estimated to be directly caused by bad AQ conditions is severely concerning [3,4]. As such, huge technological efforts and political actions, such as the Clean Air Act, are devised to improve AQ, sometimes obtaining significant impacts at regional scale. Despite some pollutants concentrations are improving due to technological advancements, nitrogen dioxide (NO_2_) and particulate matter concentrations continue to be concerning due to both their morbidity capacity and for their tendency to accumulate in several conditions (e.g., urban canyons, Pianura Padana, etc.), posing a threat to regional or block-scale communities and determining environmental iniquity for citizens [4,5]. 

Sources of air pollution range from centralized (e.g., power generation stations, industrial installations), to loosely distributed (highway traffic), to highly pervasive (road traffic, heating devices, etc.). In order to adequately plan and validate identification and remediation actions including, e.g., intelligent traffic management and/or increased public awareness, it is of paramount importance to achieve pervasive, comprehensive, and quantitative AQ knowledge [1]. Networks of highly accurate regulatory air quality monitoring stations (AQMS) are deployed all over the world by deputed environmental protection agencies (EPAs) according to precise regulatory frameworks that guide them to select deployment locations, target gases, and instrumentation technologies. The resulting reference grade instruments are usually cumbersome and costly, necessitating adequate and continuous maintenance to keep up with regulatory requirements. Cost considerations and location requisites very often lead to sparse networks that are unable to cope with the highly spatial and temporal variability of the phenomena observed in urban environments [6]. High-resolution AQ monitoring and newly arising applications such as exposome monitoring (personal exposure), and source apportionment hence needs pervasive AQMS networks that can only be deployed by resorting to low cost and sometimes portable microsensor-based devices [5]. These devices are usually connected through cellular networks of different generations or ad hoc networking structures to backend systems in which data processing takes place. Their small dimensional footprint, low cost, and low energy demands allow for truly pervasive mobile or autonomous deployments. Unfortunately, microsensors, far from being perfect gas sensors, are usually affected by several error sources, among which interference from non-target gases, environmental influences, and sensor aging/pollution are the worst ones [5]. In the long run, they eventually cause the degradation of any calibration algorithm accuracy when forced to infer concentrations in conditions which differ from calibration conditions [7]. Novel low-cost particulate matter (PM) sensors, although generally reported to achieve good accuracy records, are also subject to environmental interference. As a result, they have to be carefully evaluated in terms of accuracy with long-term field deployments, and the calibration procedure takes a paramount role in determining the overall performance and meeting demanding data quality requirements [8]. On the other hand, most devices available on the market, and particularly those devised for the consumer market, are sold without any accuracy warranty. As such, their data can be considered of limited use for most of our applications. Optimal calibration procedures are actively investigated, with field data emerging as the primary source of information to obtain an adequately accurate calibration function capable to estimate pollutant concentrations from a raw sensor signal, while correcting for the multiple factors noted above [9]. Yet, laboratory calibration is a fundamental source of information on sensor behavior and allows for a controlled appraisal of the different source of interferents and linearity characteristics of the single sensors. Researchers are still trying to reduce costs of field or laboratory calibration procedures by resorting to so-called network procedures, reducing the amount of field recorded samples to achieve an adequate calibration quality. Machine learning is actually extensively exploited to achieve best performances; several algorithms have been proposed for multisensors and soft calibration (see [10]) with mixed results and without the emergence of a winning paradigm [11]. At the same time, mid- and long-term deployment experimentations are carried out to assess multisensor devices over time. In these settings, multilinear regression, shallow neural networks, and random forests seem the most convincing approaches, having been applied with positive outcomes [11,12,13].

Citizen and unskilled personnel involvement is currently mandatory to improve AQ awareness and achieve pervasivity requisites. Many research projects have been funded and started with the declared objective of boosting awareness and enabling personal exposure monitoring in the framework of citizen science campaigns [14]. Crowdfunding and crowdsensing campaigns may in fact help to involve citizens from the beginning of a pervasive sensing project, while keeping the commitment to sufficient levels during monitoring campaigns [15,16,17].

In contrast, the amount of data generated from such a pervasive and hybrid network of mobile and fixed devices can be significant while the number of citizens and different operators that are interested in different products that can be developed by data processing is constantly growing. Actually, the need for the integration of such intelligent multisensory devices into an IoT infrastructure is perceived as very urgent. To answer to these emerging needs, beginning in 2005 our group joined the efforts to develop technological procedural and algorithmic technology to sustain the development of pervasive air quality monitoring networks [18]. 

In this work, we report the results of the design, development, and validation of an IoT AQMS architecture called MONICA (MONItoraggio Coooperativo della qualità dell’Aria—an acronym that can be translated into “Cooperative Air Quality Monitoring”). Aimed toward the development of a comprehensive (fixed, mobile regulatory) and participative air quality monitoring network (AQMN), the MONICA architecture is based on a hybrid network including portable low-cost devices relying on arrays of electrochemical sensors and calibrated particle counters. We started the project with a crowdfunding campaign that allowed us to design, build, and functionally validate prototypes of a low-cost air quality monitoring device (AQMD). Further improvements in fine-tuning the device and development of ad hoc calibration and exposure monitoring procedures were carried out during the CONVERGENCE project, leading to the current version being the starting point and the main technological enabler of an EU-funded urban innovation project, called AirHeritage, targeted to air quality monitoring and improvement in small and medium dimension but highly populated cities [15]. Most recent results include the field accuracy validation in semicontrolled, co-location experimental deployment, and functional validation in a crowdsensing campaign performed during COVID-19 lockdown phase 2 in Italy. Section 2 provides details on the MONICA architecture and the methodological and preparation aspects of the validation campaigns. Section 3 then includes the results with a particular focus on the characterization and long-term accuracy assessment of the device, and the fusion of field-recorded opportunistic data coming from a crowdsensing campaign.

## 2. Materials and Methods

### 2.1. The Monica Architecture

MONICA brings into its name its main purpose. The aim of the whole system is to make air quality assessment possible by means of several cooperative devices, either moving or fixed, distributed in a specific geographic area. Such a system requires three domains of development. 

The first domain concerns the sensing nodes; these are designed to take accurate measurements of the concentration of pollutant gases in the air in the surroundings of pollutant emission targets (power generation stations, roads, heating devices, industries). In the next section, the hardware that build the node are presented in detail; here it is worth noting that an important role of the node is the transmission of the measurements to a second tier that is the network responsible for collecting all of the data coming from every single node. 

The second development domain takes care of the network in charge of collecting the data sent by the nodes, and the backend that transforms rough data into information comprehensible to the user. At this stage, several solutions can be adopted. In the next sections, the technique used in this project is presented in detail. 

The third development domain concerns the presentation of the results in a form that is quickly human comprehensible and enables citizens (the users) to make informed choices on their behavior. Because of its large diffusion and ease of use, a smartphone was chosen as the means to present the results of the measurements to the users.

#### 2.1.1. The MONICA Node

**Figure 1:**
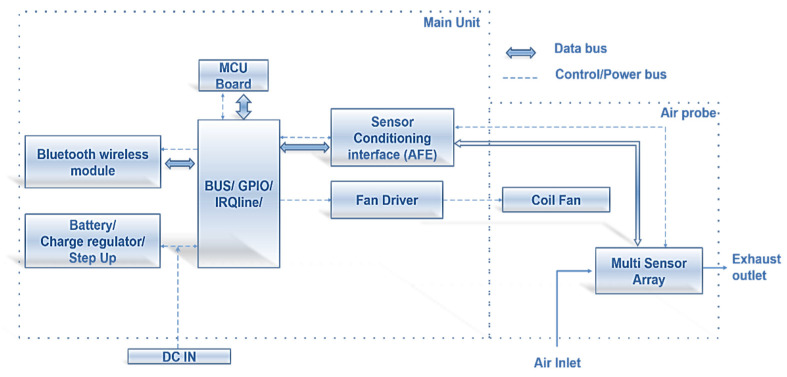
MONICA 2.0 (CONVERGENCE project version) node architecture.

As noted, MONICA is based on sensor nodes to capture periodic measurements of the concentration of harmful gases in the air. The basic device is composed of the elements shown in Figure 1.

The node was designed from scratch with the goal to keep its power consumption at reasonable levels, overcoming battery duration limits and achieving recharge times that are compatible with its smartphone counterpart. All of the components of the board have low-power characteristics including the electrochemical sensors at its core. Whenever possible, all of the unused components are put to sleep conditions.

The power supply is provided to the entire node by a board equipped with a 3.7 V battery, a battery charger, and a step-up converter that boosts the voltage to 5 V. The battery has a capacity of 3800 mAh and when the node is driven in low power mode, it can stay in operation without recharge for more than 20 h.

The main board is equipped with an array of three Alphasense sensors: CO (carbon monoxide) (CO-A4), NO_2_ (nitrogen dioxide) (NO_2_-A43F), and O_3_ (ozone) (OX-A431) and it is ready for volatile organic compounds (VOCs) measurements when the specific photoionization device (PID) sensor is installed. These sensors output a voltage signal related to the concentration of the specific gas. The sensors are mounted on an analog frontend (AFE 810-0020-00) that output some signals related to the concentration of gases in the air. The signals coming from the analog frontend are acquired and converted by a Nucleo LK432KC board from ST Microelectronics and equipped with an STM32 microcontroller with an integrated 12 bit ADC for the acquisition of the signals. The node is also capable of measuring temperature and humidity, through a digital temperature/humidity sensor located in proximity of the AFE, which communicates with the microcontroller by means of a serial interface. The information returned by the sensor allows for partially accounting for the environmental conditions, which affect the gas concentration measurements; thus, if the operating conditions are known, some appropriate corrections can be made by the firmware. The input analog signals are slowly variable so that a relatively low sampling frequency can be used, enabling the microcontroller to execute further tasks during its operation. The ADC resolution (and range) allows for the acquisition of samples representative of concentrations that are significantly higher than required for typical air monitoring devices (0–500 ppb). In the special case of CO, it is possible to sense values in the entire sensor range extension (0–12 ppm).

**Figure 2:**
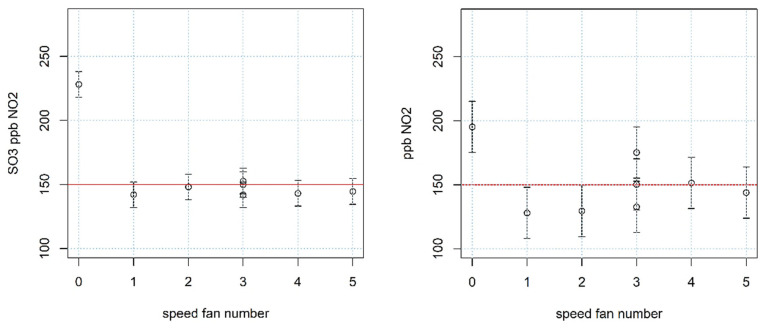
Estimation of the concentration and confidence intervals for NO_2_ using an O_3_+NO_2_ sensor and NO_2_ sensor, both exposed at a fixed concentration of 150 ppb NO_2_.

The node is equipped with two fans that can be operated at five different speeds. The forced ventilation guarantees the minimum air flux to the sensors to follow the concentration dynamics and fosters the reactivity of the system. Experimental trials with MC36304 fans were executed at different speeds. All of the tests were conducted exploiting a sensor conditioning chamber with a fixed concentration of 150 ppb of NO_2_ (which is a frequently encountered concentration while in field), varying the rotation speed of the fans in a range of five values. The gas chosen for the trials is NO_2_ because it is the most sensitive to problems related to the air flux. The sensors used for the detection are of the same type as those mounted on the nodes. The results are shown in Figure 2.

These trials showed that the used sensors need a minimum flow of air to properly work at the nominal operating conditions, and allowed for tuning the speed of the fans to the minimum in order to save energy while guaranteeing good performance of the sensors. The performance of the sensors varies at different air flow conditions. Some gases (e.g., NO_2_ and O_3_) are difficult to blend with air and are not very stable in the case of sensors enclosed in partially open chambers; the fan rotation not only fosters the blending but it also compensates the problem of instability with the injection of more air.

After detection, conditioning, sampling, and digital filtering, the microcontroller stores in its registers a set of values that encode the information needed to estimate the concentration of CO, NO_2_, O_3_, and possibly VOCs in the air. These values are the input of the second stage of the MONICA data path where a calibration algorithm infers the actual concentration.

A simple factory data-based algorithm is implemented onboard, relying on typical linear calibration function for this type of EC sensor:

(1) $$Concentration\;\left(ppb\right)=\frac{1}{S}\left[\left(Vwe_{measured}-Vae_{measured}\right)-\left(Vwe_{zero}-Vae_{zero}\right)\right]$$

using factory or laboratory computed sensitivity S (mV/ppb) and the offset Vwe_zero and Vae_zero sensor signals (electronic and zero clean air sensor offsets in mV).

Raw and factory calibrated data need to be transferred to the second stage of the system. The strategy chosen for this purpose is to exploit the connectivity capabilities of a smartphone (SP) for data transfer. The measured values are transferred from the node to the SP via Bluetooth (BT). The HC06 BT transceiver was installed on the board; it communicates with the microcontroller by means of a serial interface and on the other side it establishes a BT connection with the SP. The communication needs to be reliable, secure, and operate in such a way that the losses of information are minimized. Thus, the node is used in association with an SP hosting an application that has three main functions: first, it associates useful information to rough data (e.g., GPS coordinates); second, it offers a user friendly interface; third, it serves as gateway toward the backend.

Special attention was required during the mechanical design of MONICA. A robust design that prevents failures due to shocks of a reasonable extent or incorrect measurements due to adverse weather conditions is essential for gathering reliable information on the gas concentration. In addition, a part of the enclosure is composed by metallic material that acts as a shield that protects the board and the EC sensors from electromagnetic interferences.

#### 2.1.2. Future Works

Using the trials conducted, the minimum value for the rotation speed that guarantees the achievement of a sufficient air flow was found. Thus, in the next version of MONICA (currently under test), the fans have a fixed speed and are driven by a transistor, which in turn is driven by a general purpose pin of the microcontroller; this makes it possible to switch off the fans when not needed, thus enabling a low-power state of the entire system.

The analog signals coming from the sensors were amplified to match the ADC span and filtered to reduce the electric noise. The sampling frequency was increased to enable digital filtering operation. The printed circuit board (PCB) was redesigned in order to minimize the electric noise to which inevitably the board is posed to.

The main improvement on the new node compared with the current version is the possibility to perform particulate concentration measurement. In particular, a Plantower PMS7003 sensor is used. It comes in a compact package that fits well in the node’s case; it communicates with the microcontroller by means of a UART interface; the effective range of the sensor is 0 to 500 μg/m^3^ and has a resolution of 1 μg/m^3^. The last improvement concerns the Bluetooth transceiver; the new one is a Bluetooth low energy (BLE), which makes it possible to further reduce power consumption and enables communication with modern smartphones, which are adopting this technology as an interface to other devices. This upgrade will be the focus of the next MONICA version (3.0).

### 2.2. Device Calibration and Validation Strategies

Here, we depict the details of some of the most relevant validation campaigns performed during the years of development of the second version of the MONICA architecture, mainly within the framework of the CONVERGENCE project. Actually, a crowdfunding campaign provided the basis for proof-of-concept and the first functional validation in an operational environment. About one year after the end of this campaign, a two-plus-year field deployment experiment, which only recently ended, began with the aim of developing appropriate calibration strategies and assessment of the performance in an operative environment of the device itself. Finally, an operative measurement campaign conducted with four MONICA devices was performed in Portici (a town located 7 km south of Naples) while Italy was enduring phase 2 of the lockdown induced by COVID-19 pandemic, which directly involved citizens. The following section depicts the results.

#### 2.2.1. Crowdfunding Campaign

The MONICA 2.0 device was the target of a crowdfunding campaign that lasted 3 months and ended on 17 December 2016 [19]. The campaign was deemed as an optimal financial tool for an internally funded project, and helped to bridge the gap between us, the researchers, and citizens, while also catching the interest of regulatory monitoring authorities.

The funds raised were used for the development of a fleet of 10 multisensor units and for their laboratory-based calibration. The citizens were divided into categories according to their contribution and received a corresponding reward in return (see Table 1).

All funders obtained access to the anonymous data registered by premium crowdfunders and participated in a newsletter campaign to be informed on the project’s development. The “Smog Hunters”, instead, participated in the functional test by receiving a MONICA device and related smartphone application for a period of one month at their premises.

The campaign was advertised on national press, national television, radio programs, and social networks. In particular, scientific television and radio programs have shown their interest toward the project, helping the campaign to reach the success expected. Moreover, the citizens’ involvement was kept high in the campaign by means of feedback and suggestion questionnaires. Personal acknowledgments to each founder were published on the Eppela web page [19].

#### 2.2.2. Laboratory Characterization and Calibration Setup

An ad hoc setup was developed in the ENEA gas sensors characterization laboratory for the purpose of implementing a characterization and first performance assessment campaign.

**Figure 3:**
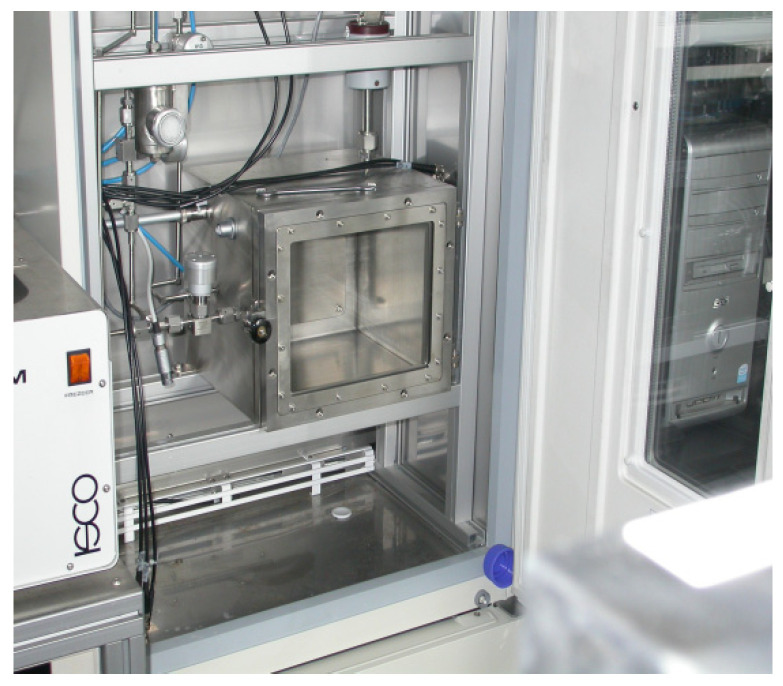
Photo of the 15 L large volume test chamber (LVTC).

A 15 L large volume test chamber (LVTC, Figure 3) was installed in a state-of-art gas sensor characterization system (GSCS). In brief, the GSCS consists of a stainless air-tight test chamber closed in an adjustable thermal box.

In the LVTC, the air composition (humidity and chemical compound concentrations) is setup by using an inlet of GAS flux precisely controlled by certified mass flow controllers (MKS 1179 series). The accuracy of the gas chemical composition is ensured by the mixing of certified bottles (Rivoira SpA). For the accuracy on the nitrogen dioxide concentration, further validation is necessary by coupling the chamber gas output to a Teledyne T200 chemiluminescent total nitrogen oxide analyzer. Temperature and humidity are recorded with industrial sensors (LSI Pt100). The LVTC can sustain the calibration of several complete sensor systems at once. The calibration method consists in injecting in the inlet tube of the LVTC a constant flow of the target gas properly diluted at the maximum concentration *(C*_0_) with humid synthetic air. The time-rising concentration *C(t)* of the target gas is precisely predicted by the following exponential law that generally describes a transition between two steady states of a physical parameter under a time constant perturbation:

(2) $$C\left(t\right)=C_{0}\left(1-e^{\frac{-1}{\tau }}\right).$$

The characteristic time (*τ*) can be precisely estimated using a calibrated sensor; this parameter is proportional to the free space inside the chamber and must be appropriately corrected when several sensors are inside the chamber.

The calibration procedure (run) consists of three time steps: first, synthetic air is injected for the unperturbed state recording of the sensor output (baseline); in the second step, the properly diluted gas target in the gas carrier is injected and the adsorbing phase of the sensor response is recorded; finally, in the third step, the test chamber is washed in a constant flow of synthetic air while recording the desorbing phase of the sensor output. With this procedure, it is possible to verify the sensor output behavior during the adsorbing and desorbing phase of the chemical compound on the surface of the sensors. Sensing hysteresis or poisoning can be detected and measured. 

With a gas flow of 1 L/min, the *τ* of the LVTC is estimated to be 1100 s ± 50 s. This means that in 3 h, several sensors with a time response faster than 2 min can be calibrated from 0 to *C*_0_ with the maximum precision allowed.

#### 2.2.3. Semicontrolled Field Conditions Setup

**Figure 4:**
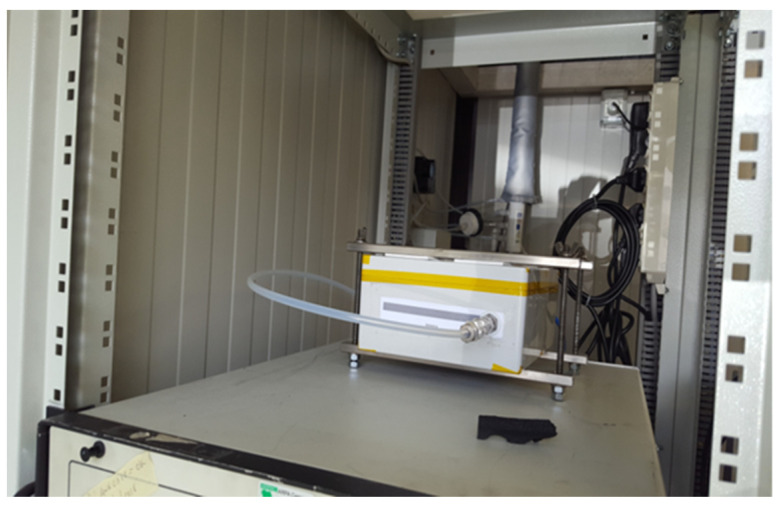
MONICA device located inside the AQMS unit showing the air-feeding scheme from the collective manifold.

On 4 April 2018, a MONICA device fully equipped with NO_2_, O_3_, and CO sensors was deployed in co-location with a regulatory AQMS located in Naples (Via Argine, codenamed NA09 in the regional inventory. The AQMS was operated and maintained under the control of the regional environmental protection agency (ARPA–Campania) [20]. The device was encased in a box a few cm larger than the device itself, and air coming from the actual station’s heated air manifold was passed through the inlet as a result of the action of a downstream rotary pump (see Figure 4). As a result, the MONICA device was analyzing the same air matrix that was fed to the regulatory grade instrument equipping the AQMS. The MONICA device was connected, using the BT connectivity, to a Raspberry Pi ver. 3 datalink, which was running RASPBIAN OS and a Python script devised to receive data from the device and provide local storage and remote transmission through an ad hoc Wi-Fi link to the MONICA backend systems. Both the Raspberry Pi and the pump were powered through an intelligent outlet that could be switched on and off remotely.

MONICA was deployed and operated within the AQMS building box unit and, as such, operating temperatures were kept in a 15 °C wide range by the unit HVAC system that remained fully functional for most of the deployment time. When analyzing these results, it should be taken into account that the temperature interference was therefore limited with respect to full outdoor fluctuations, and its associated performance loss. This setup, however, could highlight potential sensor aging or poisoning effects, making it easier to compare the sensor responses over similar environmental and target gas concentration conditions. The inlet air temperature variation was kept at a minimum by the HVAC, notwithstanding external conditions with relative humidity depending on outside absolute humidity.

#### 2.2.4. Long-Term Semicontrolled Field Calibration Dataset and Procedures

Relying on this setup, a co-location campaign was performed, exceeding 2 years of total duration. To assess performance, including the test of a more recent but costly adaptive calibration scheme, the recorded dataset allowed for different investigations that implemented several calibration strategies [21]. 

**Figure 5:**
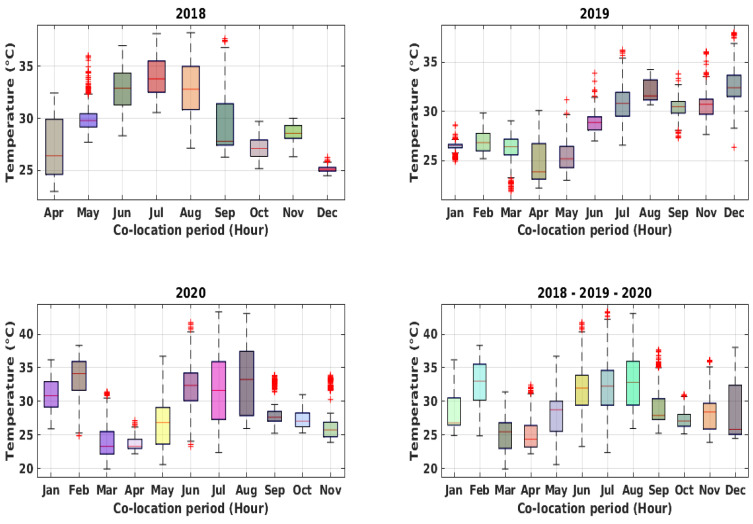
Box plots of hourly recorded temperature during the entire co-location period and for each year.

**Figure 6:**
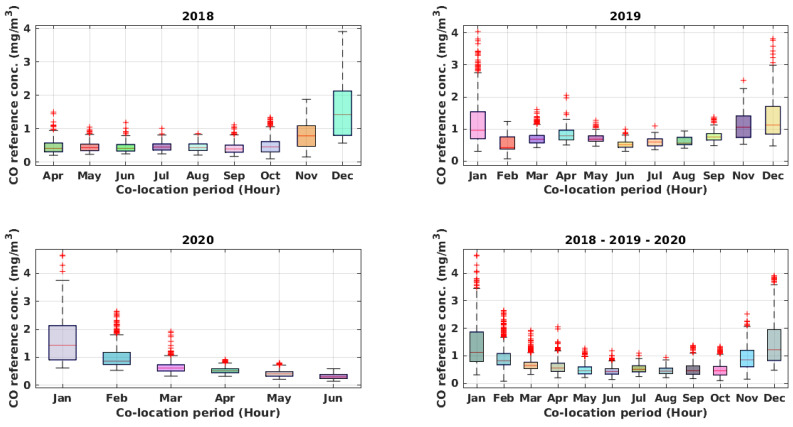
Boxplots of hourly recorded CO reference concentration during the entire co-location period and for each year.

**Figure 7:**
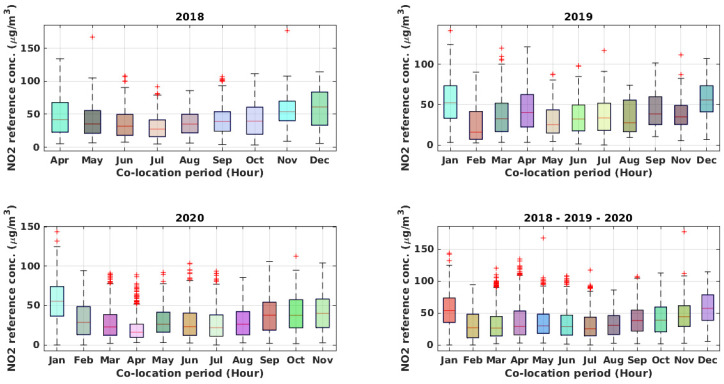
Boxplots of hourly recorded NO_2_ reference concentration during the entire co-location period and for each year.

The recorded dataset consists of 13,600 hourly samples recorded from April 2018 to November 2020. As a result of the COVID-19 pandemic, a significant reduction of pollutant concentrations could be observed during 2020, particularly from March to June and during November 2020, compared to the corresponding months of the previous years. During the co-location period, notwithstanding the presence of an air conditioning (AC) unit, the inside of the reference station underwent significant temperature oscillations, which occasionally peaked (Figure 5) to more than 40 °C. This was due partially to an incorrect set-point of the HVAC system and to its malfunctions. Specifically, the dataset contains the hourly averaged data from the device, i.e., working electrode (WE) and auxiliary electrode (AE) raw sensors readings (mV) for NO_2_, CO, O_3_, plus temperature (°C) and humidity (%), joined to hourly averaged data from the ARPAC reference analyzer for CO (ppm) and NO_2_ (ppb) (see Figure 6 and Figure 7). The dataset was preprocessed, removing any record containing missing values, and detecting and removing possible outliers with common 6-sigma threshold-based procedures.

Next, the calibration procedure was implemented using two different multivariate ML methodologies: multiple linear regression (MLR) and a three-layer shallow neural network (SNN) using three sigmoidal tangent neurons in the hidden layer and linear output neuron. The algorithms use as input raw sensor data together with environmental variables to correct the interferences and return concentration estimations as output:

(3) C=f(X), 

with

(4) $$C=\left[\begin{array}{c} C_{1}\\ C_{2}\\ \begin{array}{c} .\\ .\\ C_{n} \end{array} \end{array}\right],$$

as the relevant pollutants concentration vector and

(5) $$X=\left[\begin{array}{c} X_{1}\\ X_{2}\\ \begin{array}{c} .\\ .\\ X_{n} \end{array} \end{array}\right],\;$$

as the input variable array using raw data from the sensors (working and auxiliary electrode voltage) for target gas and interferents, plus environmental interferents. In our case, multiple algorithms were optimized to estimate a single pollutant concentration, so C represented either NO_2_ or CO concentrations.

Actually, for NO_2_, temperature readings along with NO_2_-targeted sensor data were used:

(6) $$X=\left[\begin{array}{c} {\mathrm{WE}}_{\mathrm{NO}2}\\ {\mathrm{AE}}_{\mathrm{NO}2}\\ T \end{array}\right].\;$$

The same applied to CO gas concentration, which was estimated using CO sensors WE and AE, plus temperature readings:

(7) $$X=\left[\begin{array}{c} {\mathrm{WE}}_{\mathrm{CO}}\\ {\mathrm{AE}}_{\mathrm{CO}}\\ T \end{array}\right].\;$$

Different choices of training and test set length were implemented in order to identify the best training set dimensions for an optimal calibration procedure. The results are shown in Section 3.2 and focus on NO_2_ and CO, while O_3_ reference data were not available. 

#### 2.2.5. Final Crowdsensing Validation Campaign and Calibration Procedures

In order to validate the crowdsensing capabilities of the device, during phase 2 of first lockdown due to COVID-19 (end of May 2020), four MONICA devices, previously field calibrated, where assigned to four citizens associations for a 15 days monitoring campaign. 

**Figure 8:**
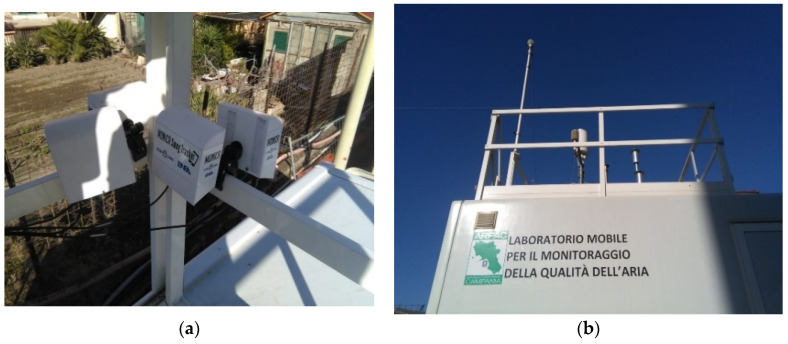
The four calibrated MONICA devices during the co-location period (**a**), with ARPAC mobile reference station (**b**) in Portici city area.

From the first of January 2020 to 1 March 2020, these four MONICA devices were collocated for 2 months with an ARPAC mobile laboratory for recording both nodes and reference measurements data (see Figure 8).

**Figure 9:**
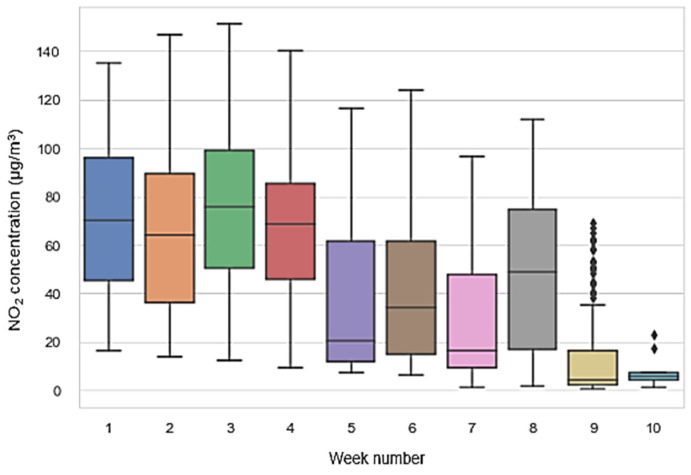
Weekly averaged concentrations of NO_2_ during co-location time.

The recorded datasets consist of 1440 h captured in a continuous sampling mode. Specifically, for each node, two datasets, with samples averaged at minute and hourly rate, have been built. Data from each of the MONICA sensor, i.e., WE and AE raw sensors readings (mV) for NO_2_, CO, O_3_ targeted sensors plus T (°C) and RH (%), were joined to same time scale averaged data from the mobile ARPAC reference analyzer for NO_2_ (µg/m^3^), CO (mg/m^3^), and O_3_ (µg/m^3^). In Figure 9, weekly averaged concentrations of NO_2_ are shown, during the co-location period.

These data were used to train linear and nonlinear (shallow neural network) models whose performances were compared in order to select an optimal calibration strategy. Assuming that *X* is the input feature vector, including WE and AE for each of the relevant sensors and *y* the predicted value, the MLR model used can be mathematically expressed by

*y* = *Xβ* + *c*, (8)

where *c* is the intercept and *β* is the least square optimal coefficients.

In addition, a shallow neural network, with three-layer architecture, empirically equipped with three standard sigmoidal tangent neurons units in the hidden layer and a linear output layer, was selected as the nonlinear algorithm. In particular, automatic Bayesian regularization (ABS) was used as the training algorithm. Since the objective of the campaign is sensor fusion of opportunistic data, in this chapter we also report the results obtained for the NO_2_ hourly averaged concentration estimation problem using hourly averaged working electrode (WE) and auxiliary electrode (AE) sensors data for NO_2_, O_3_, and CO sensors plus temperature and humidity data as inputs for the two calibration algorithms. For both algorithms, the input matrix X therefore included eight features (WE_NO_2_, AE_NO_2_, WE_CO, AE_CO, WE_O_3_, AE_O_3_, T, and RH). The two calibration algorithms were compared using different training lengths; the remaining data were used for testing purposes to simulate real conditions where nodes would have been operated after the calibration took place.

**Figure 10:**
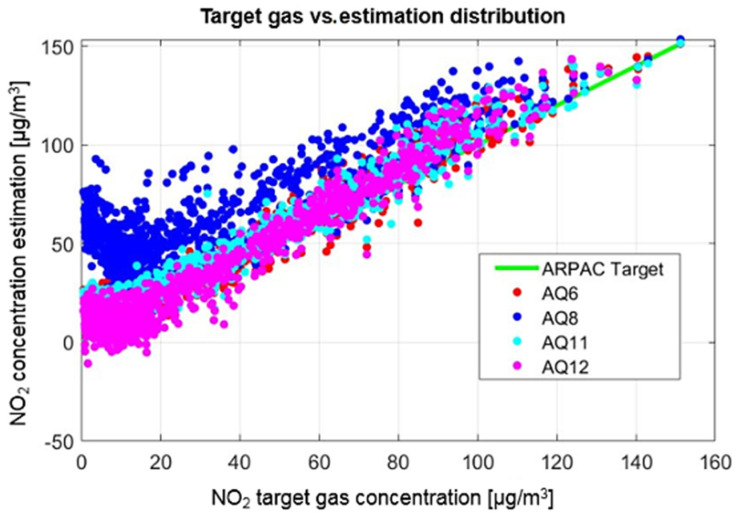
NO_2_ gas concentration estimation output using the MLR algorithm, computed for each node, along the entire co-location period vs. target gas concentration line. The performance difference among the four sensors becomes more evident when considering the low true concentration of the target pollutant; AQ8 shows a strong bias.

Analyzing the performance indicators (Table 2) for results, it is clear that limited benefit could be obtained for using more than 3 weeks of data and that MLR and NN held very similar results. In contrast, significantly different results were obtained by the four different devices with the AQ8 station standing out for its worse results. We finally chose to select the MLR algorithm as the final calibration function for all of the devices, embedding the resulting coefficient in the MONICA device-controlling Android app. In fact, we used the entire dataset for training purposes, expecting a MAE for NO_2_ estimation ranging from 6 to 12 µg/m^3^ depending on the MONICA node. In Figure 10, NO_2_ gas concentration estimation output using the MLR algorithm, computed for each node, along the entire co-location period vs. target gas concentration line is shown.

**Figure 11:**
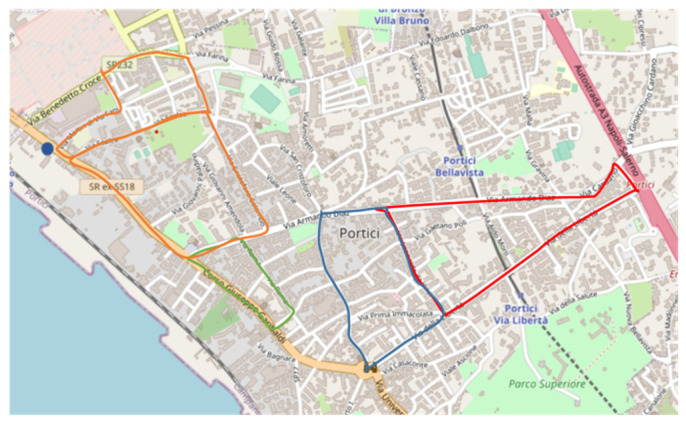
The four preset monitoring paths (red, blue, green, orange) showing the mobile laboratory location (blue dot).

The four associations that were involved selected six volunteers who used the four calibrated MONICA devices to monitor air quality according to a specific proposed monitoring scheme (Figure 11). The volunteers were trained via remote live sessions and short educational videos. This implied a minimum of 1 h cumulative duration monitoring session each working day, covered by foot and following one of four different paths, using one of the four calibrated devices that was assigned to a single volunteer on weekly basis. Each device was used on a single path. Aside from technical difficulties, only four of the total 60 (15 × 4) anticipated monitoring slots were deserted.

Data were captured and sent to the backend where a specific dataset was prepared, which included all of the available measurements. Sensor fusion was conducted using geostatistical interpolation, specifically relying on the inverse distance weighting (IDW) algorithm [22,23]. IDW was actually used to compute an average interpolation of pollutant concentrations on a predetermined grid by applying the opportunistic measurement taken in a particular time slot (in this case, the campaign duration) using:

$$c\left(x,y\right)=\frac{\sum_{1}^{N}w_{i}c\left(x_{i},y_{i}\right)}{\sum_{1}^{N}w_{i}}\;$$

where *c*(*x,y*) is the concentration at interpolated location *p* = (*x,y*), *c*(*x_i_,y_i_*) is the concentration at interpolating locations (measurement points) *p_i_* = (*x_i_,y_i_*)*,* and

$$w_{i}=\frac{1}{d{\left(p,p_{i}\right)}^{k}}$$

where *d* is the Euclidean distance with *k* = 2. At an interpolating position, IDW uses the actual concentration recordings. A preliminary step is undertaken to average all measurements that took place within a specific grid cell. Grid dimension was set at 5 m.

## 3. Results

### 3.1. Laboratory Calibration Results

Our LVCT allowed us to place up to eight of these sensor systems (Figure 8a). A Raspberry Pi 3 with Raspbian and a Python script collected data via MONICA’s parsers in the log files.

**Figure 12:**
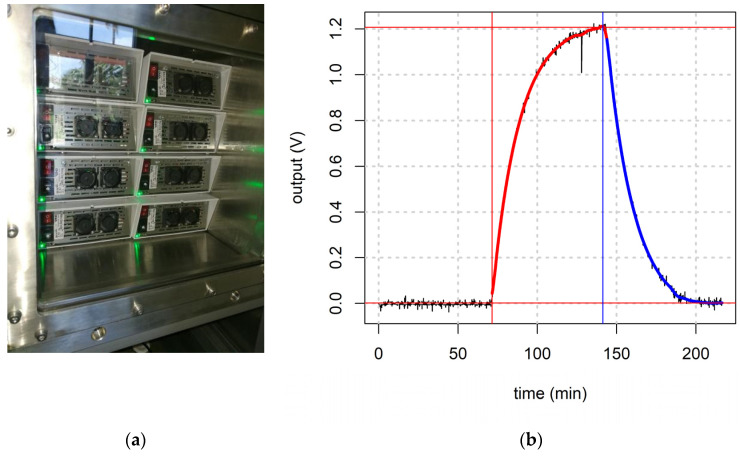
(**a**) Eight MONICA wireless sensor systems for air pollution monitoring during the calibration run. (**b**) Time log graph for one sensor output during an injection of 5 ppm of carbon monoxide. The red line shows the adsorbing phase and the blue line shows the desorbing phase of the sensor output.

**Figure 13:**
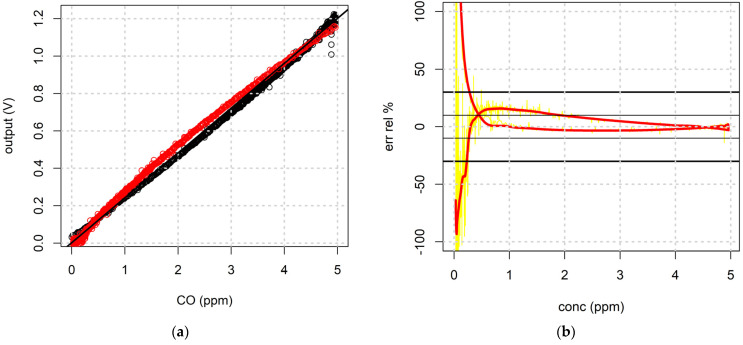
(**a**) Sensitivity curve and the linear regression for the sensor output in the range 0:5 ppm during adsorption (black dots) and desorption (red dots). (**b**) Graph of the relative error for the estimated gas concentration by the sensor output with the calculated sensitivity vs. the gas concentration.

Sensor calibration was performed vs. 0:500 ppb of NO_2_ and 0:5 ppm of CO at a controlled and constant temperature and humidity. Figure 12b shows a graph of the time log for a sensor output during a calibration run with an injection of 5 ppm carbon monoxide. It is easy to distinguish the three steps of the calibration run; the red line underlines the adsorbing phase while the blue line shows the desorbing phase. As a result of the calibration run, a sensitivity curve was estimated by the sensor output log using a script in R language that synchronizes and correlates sensor output with the gas concentration. Once estimated, the sensitivity shown with a linear regression of the data (Figure 13a), can be used to explore the precision of the sensor output in the entire range of calibration, as illustrated in Figure 13b, where the relative error of the sensor-estimated gas concentration vs. the gas concentration is reported. In this way, it is possible to estimate useful sensor parameters such as LOD (limit of detection), LOQ (limit of quantification), output linearity, precision, and accuracy.

### 3.2. Crowdfunding Results

At the end of the campaign, the MONICA project was funded for 8730 EUR, reaching 145% of the expected 6000 EUR ceiling. The campaign was fully implemented and exceeded the funding targets set with 102 collaborators. There were 44 smog hunters who used a MONICA device for 30 days together with its Android app.

**Figure 14:**
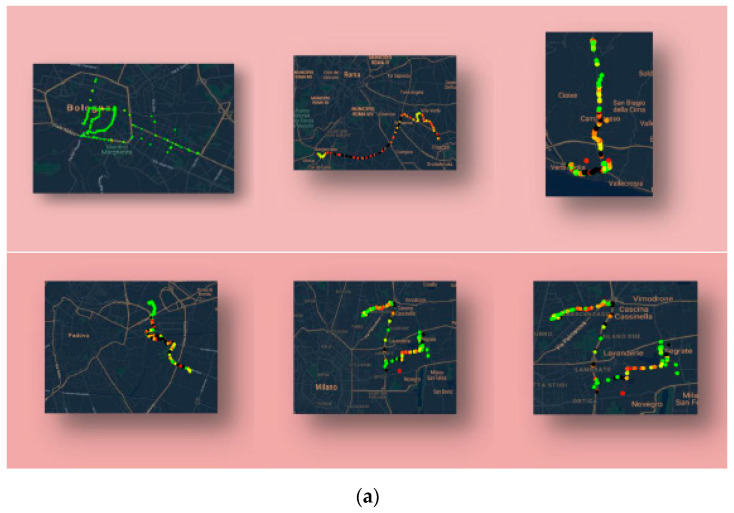

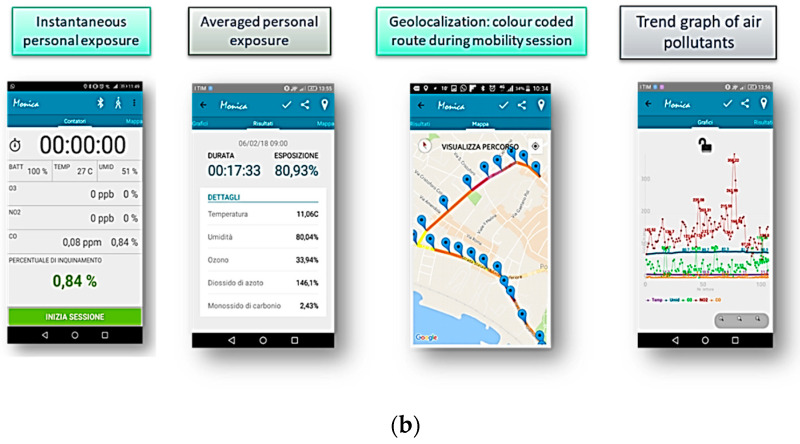
(**a**) Examples of routes maps performed by Smog Hunters (crowdfunders). The colors encode for normalized CO pollutant concentration estimation with factory-based calibration as captured by the MONICA app and (**b**) stored in the proprietary IoT backend. Note that the text is reported in Italian language in the smartphone app screenshots.

The devices were shipped to Italian crowdfunders starting in January 2018 and returning to the ENEA Laboratory at the end of the 30 days period to be sent back to the remaining users in a round-robin fashion (see Table 3). A detailed instruction manual was delivered together with the MONICA system. This operating system also made it possible to test the calibrated platform in the field as a backend. In addition, their data helped to create a significant air pollution database on which to build maps, including their routine routes in their cities (see Figure 14).

### 3.3. Two-Plus Years of Semicontrolled Field Deployment Results

In order to assess the midterm performance calibration procedures, the dataset was initially split into two training periods highlighting both seasonality effects on the empirical probability distributions of the pollutants and the environmental parameters.

For CO and NO_2_ concentration estimation, the experiments were performed by selecting different training set lengths and testing the performance on the remaining weeks. Results were cross-validated using disjoint training sets with the first fold starting from the initial week of the co-location period.

However, for CO results, the dataset was further truncated in June 2020, due to sensor malfunction.

Table 4 and Table 5 show the results for linear and nonlinear calibration procedures for CO and NO_2_ concentration estimation, respectively, computed by averaging the performance indicators on the weekly test sets. Specifically, parts a and b depict the results obtained in two different time segments of the dataset: from April 2018 to June 2019 and from July 2019 to November 2020. 

**Figure 15:**
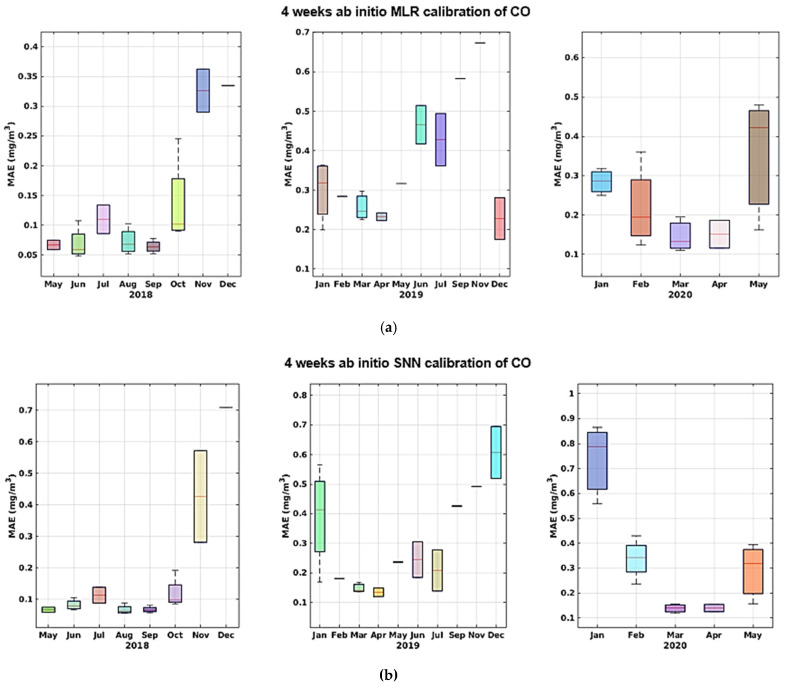
MAE trends shown by monthly boxplot for ab initio calibration of CO for MLR (**a**) and SNN (**b**); the latter shows slightly better figures during the first and last year.

**Figure 16:**
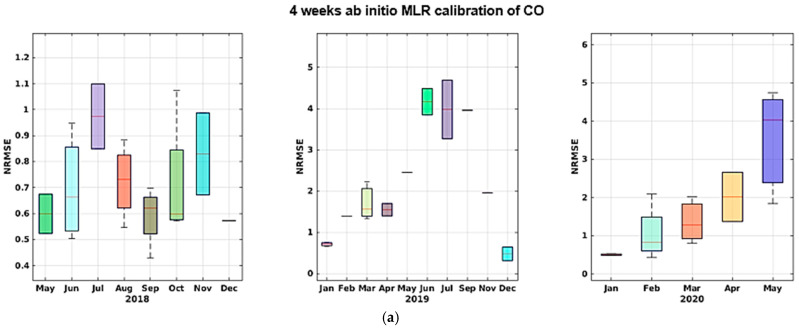

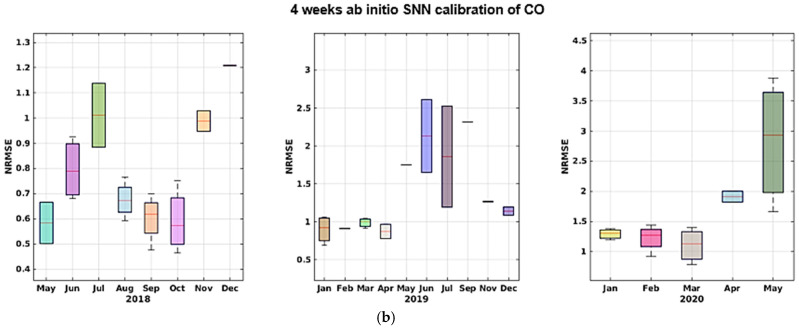
NMRSE trends shown by monthly boxplot for ab initio calibration of CO for MLR (**a**) and SNN (**b**) with the latter showing slightly better figures during first and last year.

**Figure 17:**
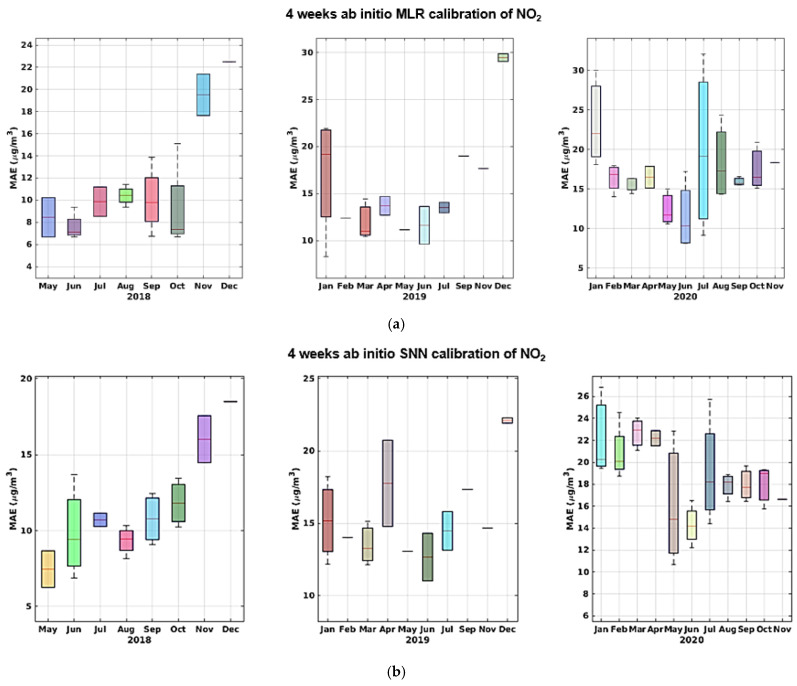
MAE trends shown by monthly boxplot for ab initio calibration of NO_2_ for MLR (**a**) and SNN (**b**); the latter shows slightly better figures consistently during the 3 years.

**Figure 18:**
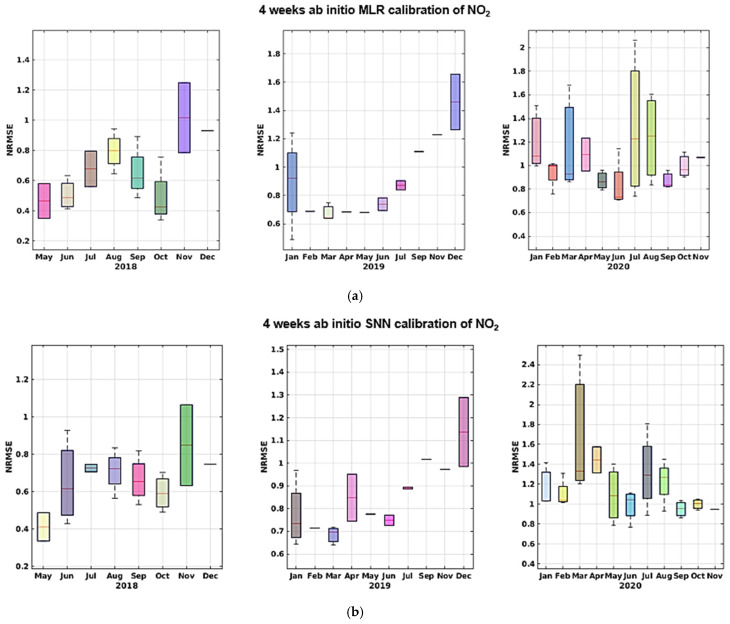
NMRSE trends shown by monthly boxplot for ab initio calibration of NO_2_ for MLR (**a**) and SNN (**b**); the latter shows slightly better figures during the first and last year.

**Figure 19:**
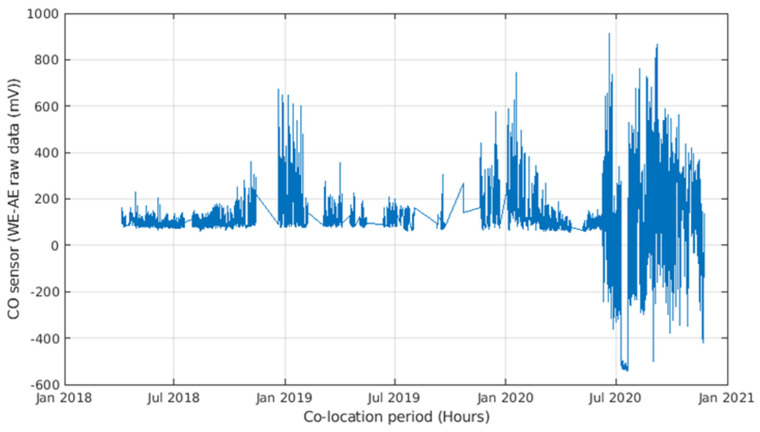
Difference between WE electrode potential and AE electrode potential (WE–AE) raw data for the MONICA CO sensor during the entire co-location period; note the sudden breakup of the sensing properties that occurred in June 2020, more than 2 years after deployment.

As we can see, the MLR calibration model almost always provides for the best calibration result in terms of MAE, MRE, and NRMSE, and shows increased efficiency in learning from sensor data in this specific configuration. Both models, however, yield good calibration results with an acceptable training-set length. More specifically, the results obtained in the two different datasets relying on the two dataset splits show no sign of sensor performance degradation. In fact, if correctly recalibrated, the performance obtained in the two halves does not show a marked worsening. In the long term, however, the performance decrease dramatically, regardless of the amount of training data, as shown by data reported in Table 4 (c,d) and Table 5 (c,d). In particular, for part c in both tables shows a cross-validated performance assessment using the largest dimension of training set considered, i.e., 4 weeks. A large performance hit is observed on NRMSE and R^2^ indicators when considering all remaining data spanning more than 2 years. Part d shows the performance assessment of using the initial 4 weeks of data of the entire dataset for training purposes. The worsening performance becomes unacceptable. Given the results shown in Table 4 and Table 5 and Figure 15, Figure 16, Figure 17, Figure 18 and Figure 19 we are forced to blame sensors and concept drift effects, which can be partially recovered by appropriate recalibration strategies.

### 3.4. Crowdsensing Validation Results

Table 6 captures the averaged concentrations as recorded by all volunteers in the entire urban territory for all four devices. For CO and NO2, the results are compatible with the expected increase in pollutant concentrations with respect to phase 1 (complete lockdown) measurements, due to the slow restart of the productive activity in the area due to the phase 2 regulatory framework. Ozone maintains similar values to those recorded during the last days of phase 1.

**Figure 20:**
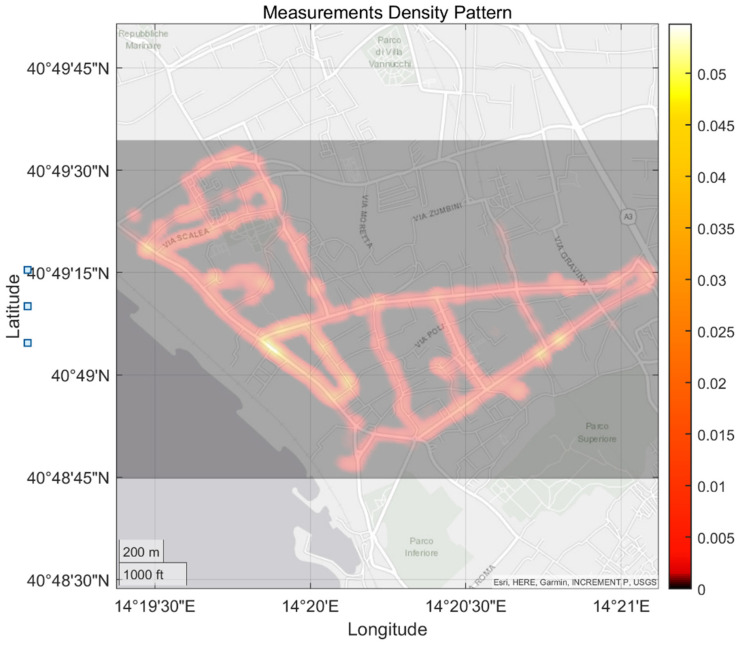
Spatial density of the opportunistic measurements sampled by campaign involved citizens. Uneven density patterns are due to the different lenghts of the preset paths. Unforeseen measurement locations are also shown due to the paths which citizens decided to take for reaching the foreseen measurement locations.

**Figure 21:**
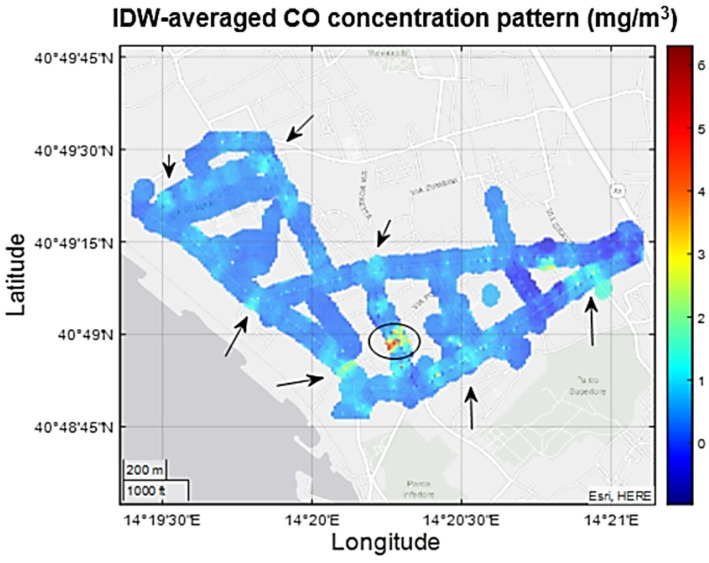
IDW-averaged CO concentration pattern is characterized by localized hotspots near main crossroads or streets characterized by heavy traffic load (arrows). Unforeseen hotspots also arose, prompting for ad hoc measurement campaigns (ellipse).

**Figure 22:**
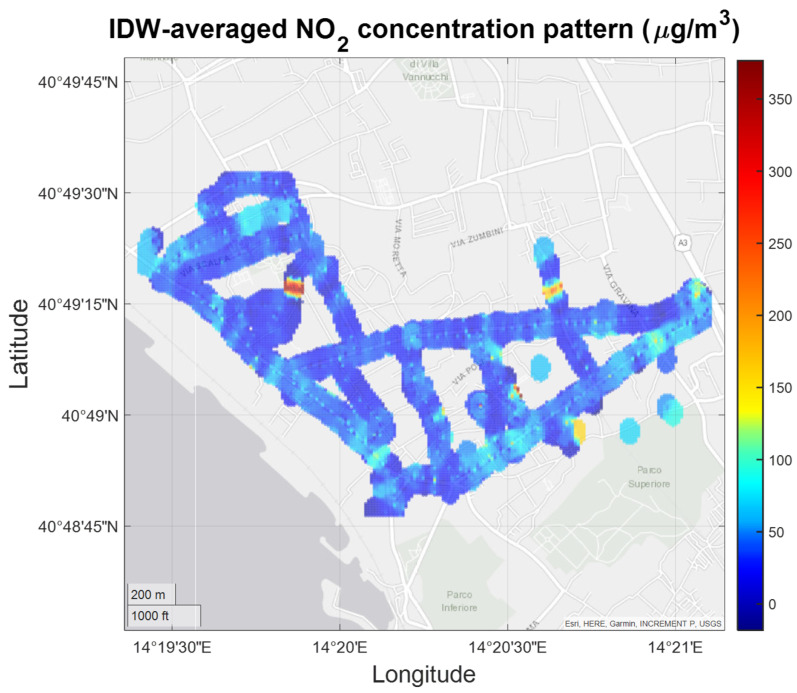
IDW-averaged NO_2_ concentration pattern confirmed hotspot areas identified by CO monitoring.

**Figure 23:**
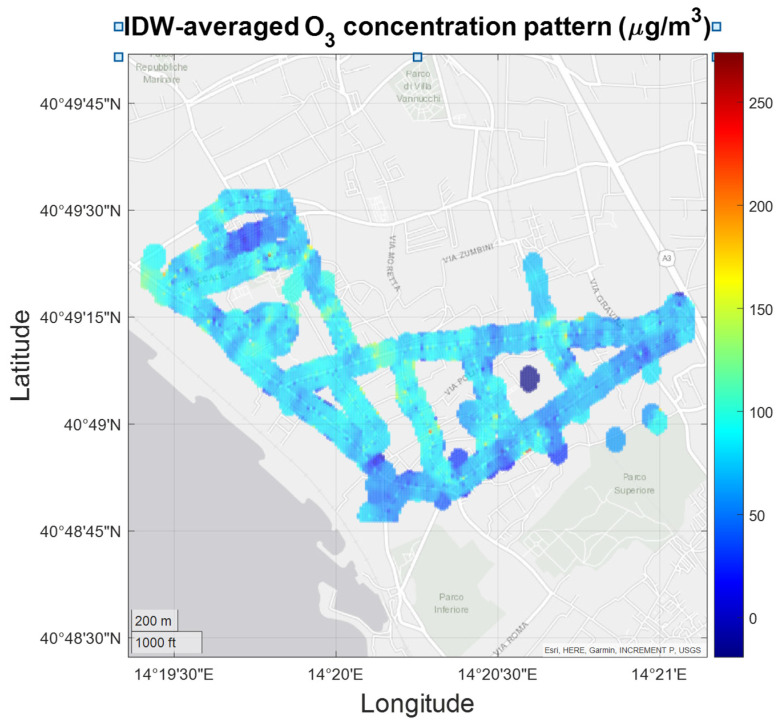
IDW-averaged O_3_ concentration pattern show generally lower spatial variance with average values that reach or overcome the regulatory threshold.

The intrinsic opportunistic nature of citizen science monitoring activities and the difference in the length of the four paths was captured by the slightly uneven measurement density computed on the recorded positioning data (Figure 20). In particular, some areas appear overrepresented due to multiple recordings taken during multiple laps over the same path. Care should take in evaluating underrepresented areas (darkest colors) that will suffer from temporal variance dependence, potentially leading to non-representative results in the IDW-averaged spatial patterns. Calibrated data featuring measured concentrations were fused to build inverse distance weighting maps. Figure 21, Figure 22 and Figure 23 show the resulting pollution patterns. Figure 21 shows the average concentration patterns of CO as monitored during the campaign from all of the volunteers, regardless of the hour of the day. These are characterized by localized hotspots located near main crossroads and in areas that are subject to heavy car traffic. However, an unforeseen hotspot emerged, confirming the unprecedented resolution power of cooperative mobile monitoring. 

NO_2_ pattern analysis (Figure 22) basically confirms the hotspots identified by CO pattern analysis; however, some of the most polluted areas are characterized by values that approach regulatory thresholds relative to measured average CO concentration values. Finally, Figure 23 shows O_3_ concentrations patterns.

Ozone IDW-averaged values show a lower spatial variance but are relatively closer to regulatory thresholds and overcome them locally. While this behavior is common during summer season in the monitored area, these results call for a closer analysis of the main drivers.

## 4. Discussion and Conclusions

In this work, an IoT architecture for high-resolution spatial and temporal air quality monitoring was developed and described. The devised architecture centers around a chemical multi-sensor system relying on electrochemical sensors and field data-driven calibration algorithms derived with machine learning approaches. A smartphone app was devised to provide real-time or delayed feedback to users while a non-relational (NOSQL) database-based website provides for data integration and fusion to the user community. Most of these advances occurred during the implementation of the FlagEra CONVERGENCE project. The multifaceted validation campaigns, including crowdfunded functional validation, laboratory characterization, long-term fixed co-location deployments, and crowdsensing, showed the viability of the project for personal, mobile, or fixed applications.

In particular, the field co-location lasted two-plus years and provided useful insights on the long-term operative behavior of electrochemical sensor arrays. In particular, we could not detect significant degradation in the potential accuracy of sensors up to more than 2 years following deployment, when one of the sensors, specifically the CO sensor, eventually broke. Notwithstanding sensors and concept drift, yearly recalibration procedures provided for recovering most of the initial year performance levels. Three weeks of co-location data with the high-accuracy regulatory-grade monitoring system showed sufficient to guarantee good performance for more than 6 months when using field calibration approaches. Long-term performance assessment with crossvalidated testing procedures, showed that, using at least 4 weeks co-location data as a training set independently from the calibration starting date, it was possible to obtain reasonably good performance on average. Performance obtained by multilinear calibration and shallow neural network were very similar with the first, providing for slightly better generalization properties.

Zooming in by using a single ex ante calibration with 4 weeks of data, we also showed how the onset of seasonal and anthropogenic variation in environmental conditions and, respectively, pollutant concentrations caused periodic worsening of accuracy that was only partially recovered when the situation returned to a condition similar to that of the calibration period. After the first year, however, the performance became totally unacceptable and a yearly recalibration routine is the minimum requirement to guarantee the performance level. 

Finally, a crowdsensing campaign showed the viability of the platform as a personal exposure device while collaboratively captured opportunistic data sharing provided for high-resolution mapping capabilities. Future work will include upscaling sensor array to include particulate matter monitoring capabilities with subsequent modification to the IoT frontend and backend subsystems, and the actual employment of the resulting architecture for a long-term full-scale crowdsensing campaign in the framework of the UIA-AirHeritage project. 

## Figures and Tables

**Table 1:** Available stakes and related premiums during the crowdfunding campaign.

Stakes (EUR)	Qualification/Reward
5	Smog Enemy: updated periodically by newsletter on project developments
10	Smog Mapper: informed about developed tests
20	Smog Tracer: accessibility to air quality maps produced by Smog Hunters
45	Smog Hunter: received MONICA 2.0 at home for 1 month
100	Smog Researcher: visitor of ENEA Laboratory for 1 day
200	Smog Patron: supporter of the project
300	Smog Master: ambassador of the project

**Table 2:** Mean absolute errors: (**a**) Mean Absolute Error, (**b**) Pearson correlation coefficient and (**c**) coefficient of Determination (R^2^) for NO_2_ estimations obtained using two calibration models with different choices for the training length (L, in weeks) for each node. Bold indicates the performance level that was best achieved.

**L**	**Mean Absolute Error (MAE) [µg/m^3^]**							
	**AQ6**		**AQ8**		**AQ11**		**AQ12**	
	**NN**	**MLR**	**NN**	**MLR**	**NN**	**MLR**	**NN**	**MLR**
1	11.7	7.94	21.94	23.36	8.20	7.78	12.23	6.55
2	7.53	7.70	25.64	16.78	10.07	9.51	8.82	6.92
3	8.89	7.73	19.48	13.30	10.09	**8.86**	8.33	6.49
4	8.74	7.56	**11.71**	12.63	10.24	9.88	7.08	6.31
5	**7.98**	**7.63**	13.15	**11.37**	**9.6**	9.65	**5.79**	**5.15**
**L**	**Pearson Correlation Coefficient r**							
	**AQ6**		**AQ8**		**AQ11**		**AQ12**	
	**NN**	**MLR**	**NN**	**MLR**	**NN**	**MLR**	**NN**	**MLR**
1	0.93	0.97	0.94	0.93	0.97	0.97	0.93	0.98
2	0.97	0.97	0.92	0.94	0.97	0.97	**0.98**	**0.98**
3	0.97	**0.98**	0.93	0.94	0.97	0.97	0.98	0.98
4	0.97	0.98	0.95	0.95	**0.98**	**0.98**	0.98	0.98
5	**0.98**	0.96	**0.96**	**0.96**	0.98	0.98	0.98	0.98
**L**	**Coefficient of Determination R^2^**							
	**AQ6**		**AQ8**		**AQ11**		**AQ12**	
	**NN**	**MLR**	**NN**	**MLR**	**NN**	**MLR**	**NN**	**MLR**
1	0.79	**0.91**	0.47	0.41	**0.91**	**0.92**	0.78	0.94
2	**0.91**	0.9	0.22	0.62	0.85	0.88	0.88	0.92
3	0.88	0.89	0.49	0.74	0.86	0.88	0.89	0.92
4	0.87	0.88	**0.77**	0.75	0.84	0.84	0.91	0.93
5	0.88	0.88	0.75	**0.81**	0.87	0.87	**0.94**	**0.95**

**Table 3:** Features of funder mobility sessions.

Monica ID	Time Period	Location	Sampling Time	No. Sessions	No. Samples
1	16 January–22 February 2018	Roma	30 s	31	2656
2	29 January–26 February 2018	Bologna	20 s	19	1129
3	19 February–16 March 2018	Padova	20 s	29	1246
4	21 February–16 March 2018	Segrate (MI)	30 s	20	774
5	19 February–15 March 2018	Novate Milanese (MI)	30 s	27	3836
6	14 March 3–27 March 2018	Sanremo (IM)	10 s	18	6150

**Table 4:** Calibration performance indicators for CO estimations obtained by using two calibration models with different choices of training length. Bold values show the best performance recorded within a single column, for median and mean (italic) of an indicator, preferring the minimum number of required samples in the case of a tie.

**(a) Cross-validated midterm CO calibration results (April 2018–July 2019).**													
		**MLR**	**SNN**	**MLR**	**SNN**	**MLR**	**SNN**	**MLR**	**SNN**	**MLR**	**SNN**	**MLR**	**SNN**
**Training Set Length**		**MAE (mg/m^3^)**	**STD**	**RMSE (mg/m^3^)**	**NRMSE**	**R^2^**	**R**	**Training Set Length**		**MAE (mg/m^3^)**	**STD**	**RMSE (mg/m^3^)**	**NRMSE**
		**MLR**	**SNN**	**MLR**	**SNN**	**MLR**	**SNN**			**MLR**	**SNN**	**MLR**	**SNN**
1 week (CV)	(Mean)	0.27	0.28	0.24	0.30	0.37	0.42	0.84	0.96	−0.06	−0.22	0.79	0.63
	(Median)	0.19	0.21	0.18	0.29	0.26	0.36	0.59	0.82	0.65	0.33	0.88	0.68
2 weeks (CV)	(Mean)	0.19	0.25	0.17	0.28	0.26	0.38	0.59	0.86	0.59	0.11	0.87	0.68
	(Median)	0.17	0.21	0.16	0.28	0.24	0.36	0.54	0.80	0.70	0.35	0.89	0.69
3 weeks (CV)	(Mean)	0.17	***0.21***	***0.16***	***0.26***	***0.23***	0.35	0.52	0.77	0.72	0.37	0.89	0.72
	(Median)	0.16	**0.20**	**0.15**	**0.26**	0.23	0.34	0.51	0.76	0.74	0.41	0.90	0.76
4 weeks (CV)	(Mean)	***0.16***	0.21	0.16	0.26	0.23	***0.33***	***0.50***	***0.74***	***0.74***	***0.43***	***0.90***	***0.74***
	(Median)	**0.15**	0.20	0.15	0.26	**0.22**	**0.32**	**0.48**	**0.71**	**0.77**	**0.47**	**0.91**	**0.77**
**(b) Cross-validated midterm CO calibration results (July 2019–June 2020).**													
**Training Set Length**		**MAE (mg/m^3^)**		**STD**		**RMSE (mg/m^3^)**		**NRMSE**		**R^2^**		**R**	
		**MLR**	**SNN**	**MLR**	**SNN**	**MLR**	**SNN**	**MLR**	**SNN**	**MLR**	**SNN**	**MLR**	**SNN**
1 week (CV)	(Mean)	0.18	0.29	0.13	0.30	0.22	0.43	0.37	0.71	0.85	0.43	***0.96***	0.85
	(Median)	0.17	0.29	0.12	0.32	0.20	0.41	0.34	0.69	0.89	0.49	0.96	0.87
2 weeks (CV)	(Mean)	***0.16***	0.24	***0.12***	0.26	0.21	0.36	0.34	0.60	0.87	0.61	0.96	0.89
	(Median)	0.16	0.22	**0.11**	0.25	**0.19**	0.35	**0.32**	0.58	**0.90**	0.64	0.96	0.90
3 weeks (CV)	(Mean)	0.16	0.22	0.12	0.23	0.21	0.32	0.34	0.53	0.87	0.67	0.96	***0.92***
	(Median)	**0.15**	0.20	0.11	0.17	0.19	0.27	0.31	0.44	0.90	0.79	**0.97**	0.94
4 weeks (CV)	(Mean)	0.16	***0.21***	0.12	***0.21***	***0.20***	***0.29***	***0.33***	***0.49***	***0.89***	***0.71***	0.96	0.92
	(Median)	0.16	**0.18**	0.12	**0.14**	0.20	**0.23**	0.34	**0.37**	0.89	**0.86**	0.96	**0.95**
**(c) Cross-validated long-term CO calibration results (April 2018–June 2020).**													
**Training Set Length**		**MAE (mg/m^3^)**		**STD**		**RMSE (mg/m^3^)**		**NRMSE**		**R^2^**		**R**	
		**MLR**	**SNN**	**MLR**	**SNN**	**MLR**	**SNN**	**MLR**	**SNN**	**MLR**	**SNN**	**MLR**	**SNN**
4 weeks (CV)	(Mean)	0.20	0.25	0.17	0.27	0.27	0.37	0.52	0.72	0.72	0.45	0.89	0.76
	(Median)	0.18	0.23	0.17	0.26	0.26	0.35	0.50	0.70	0.75	0.53	0.91	0.77
**(d) CO calibration ab initio results (April 2018–June 2020).**													
**Training Set Length**		**MAE (mg/m^3^)**		**STD**		**RMSE (mg/m^3^)**		**NRMSE**		**R^2^**		**R**	
		**MLR**	**SNN**	**MLR**	**SNN**	**MLR**	**SNN**	**MLR**	**SNN**	**MLR**	**SNN**	**MLR**	**SNN**
4 weeks	(Mean)	0.22	0.24	0.21	0.22	0.30	0.33	1.16	0.99	−0.86	−0.17	0.76	0.66
	(Median)	0.21	0.17	0.21	0.14	0.31	0.23	0.72	0.95	0.47	0.04	0.85	0.68

**Table 5:** Calibration performance indicators for NO_2_ estimations obtained using two calibration models with different choices of training length.

**(a) NO_2_ calibration with cross-validation (CV) (April 2018–July 2019).**														
**Training Set Length**			**MAE (µg/m^3^)**		**STD**		**RMSE (µg/m^3^)**		**NRMSE**		**R^2^**		**R**	
			**MLR**	**SNN**	**MLR**	**SNN**	**MLR**	**SNN**	**MLR**	**SNN**	**MLR**	**SNN**	**MLR**	**SNN**
1 week		(Mean)	16.91	15.64	14.59	12.92	22.35	20.33	0.92	0.84	−0.15	0.20	0.72	0.69
		(Median)	13.85	13.73	12.22	11.97	18.40	18.29	0.76	0.76	0.42	0.43	0.78	0.74
2 weeks		(Mean)	13.90	14.89	12.08	13.00	18.43	19.79	0.76	0.81	0.40	0.25	0.76	0.69
		(Median)	13.80	13.61	***11.23***	11.88	17.87	17.93	0.74	0.74	0.46	0.44	0.79	0.74
3 weeks		(Mean)	14.42	13.85	12.98	12.30	19.42	18.55	0.80	0.76	0.23	0.39	0.76	0.72
		(Median)	**12.81**	12.85	10.92	11.28	**16.84**	16.98	0.69	0.70	**0.52**	0.51	0.79	0.75
4 weeks		(Mean)	***13.02***	***13.34***	11.40	***11.92***	***17.33***	***17.91***	***0.71***	***0.73***	***0.49***	***0.42***	***0.78***	***0.74***
		(Median)	13.33	**11.87**	**10.69**	**10.50**	17.03	**15.80**	**0.70**	**0.65**	0.51	**0.58**	**0.80**	**0.78**
**(b) NO_2_ calibration with cross-validation (CV) (July 2019–November 2020).**														
**Training Set Length**			**MAE (µg/m^3^)**		**STD**		**RMSE (µg/m^3^)**		**NRMSE**		**R^2^**		**R**	
			**MLR**	**SNN**	**MLR**	**SNN**	**MLR**	**SNN**	**MLR**	**SNN**	**MLR**	**SNN**	**MLR**	**SNN**
1 week		(Mean)	18.04	18.19	14.12	13.94	22.92	22.94	0.99	0.99	−0.04	−0.05	0.60	0.54
		(Median)	16.30	16.80	12.78	12.96	20.96	21.20	0.90	0.92	0.18	0.15	0.65	0.58
2 weeks		(Mean)	16.13	17.73	12.63	13.62	20.50	22.39	0.89	0.97	0.19	−0.01	0.63	0.56
		(Median)	15.59	17.11	12.10	13.12	19.79	21.68	0.86	0.94	0.27	0.11	0.68	0.60
3 weeks		(Mean)	15.20	17.06	12.05	13.78	19.41	21.95	0.84	0.95	0.27	0.04	0.66	0.56
		(Median)	14.46	15.71	11.55	12.65	19.01	20.06	0.83	0.87	0.32	0.24	0.68	0.63
4 weeks		(Mean)	***13.76***	***14.73***	***10.99***	***11.83***	***17.62***	***18.90***	***0.76***	***0.82***	***0.41***	***0.31***	***0.71***	***0.65***
		(Median)	**13.96**	**14.59**	**10.99**	**11.53**	**17.74**	**18.53**	**0.77**	**0.80**	**0.41**	**0.35**	**0.71**	**0.66**
**(c) NO_2_ calibration with cross-validation (CV) (April 2018–November 2020).**														
**Training Set Length**	**Test Set Length**		**MAE (µg/m^3^)**		**STD**		**RMSE (µg/m^3^)**		**NRMSE**		**R^2^**		**R**	
			**MLR**	**SNN**	**MLR**	**SNN**	**MLR**	**SNN**	**MLR**	**SNN**	**MLR**	**SNN**	**MLR**	**SNN**
4 weeks	4 weeks CV	(Mean)	15.09	16.55	12.40	13.96	19.54	21.67	0.82	0.90	0.32	0.12	0.70	0.61
		(Median)	14.91	15.59	12.08	12.91	19.41	20.61	0.81	0.86	0.34	0.25	0.72	0.66
**(d). NO_2_ calibration ab initio (April 2018–November 2020).**														
**Training Set Length**	**Test Set Length**		**MAE (µg/m^3^)**		**STD**		**RMSE (µg/m^3^)**		**NRMSE**		**R^2^**		**R**	
			**MLR**	**SNN**	**MLR**	**SNN**	**MLR**	**SNN**	**MLR**	**SNN**	**MLR**	**SNN**	**MLR**	**SNN**
4 weeks	4 weeks	(Mean)	14.72	15.68	11.22	10.78	18.56	19.07	0.86	0.89	0.18	0.11	0.69	0.60
		(Median)	14.93	15.83	10.78	10.95	17.41	19.20	0.84	0.88	0.28	0.22	0.70	0.62

**Table 6:** First-order characterization of recorded data.

	First-Order Statistics	
	Average	Standard Deviation
CO (mg/m^3^)	0.44	0.64
NO_2_ (µg/m*3*)	40.0	37.1
O_3_ (µg/m^3^)	76.2	34.3

## Data Availability

Supporting data are currently available on request and are expected to be made publicly available during 2022.
